# Bispectral index to guide induction of anesthesia: a randomized controlled study

**DOI:** 10.1186/s12871-018-0522-8

**Published:** 2018-06-15

**Authors:** Dirk Rüsch, Christian Arndt, Leopold Eberhart, Scarlett Tappert, Dennis Nageldick, Hinnerk Wulf

**Affiliations:** Department of Anesthesia and Intensive Care, University Hospital Giessen-Marburg, Marburg Campus, Baldingerstraße, 35033 Marburg, Germany

**Keywords:** Anesthesia, Induction, Propofol, Hemodynamics, Hypotension, Electroencephalogram, Bispectral index

## Abstract

**Background:**

It is unknown to what extent hypotension frequently observed following administration of propofol for induction of general anesthesia is caused by overdosing propofol. Unlike clinical signs, electroencephalon-based cerebral monitoring allows to detect and quantify an overdose of hypnotics. Therefore, we tested whether the use of an electroencephalon-based cerebral monitoring will cause less hypotension following induction with propofol.

**Methods:**

Subjects were randomly assigned to a bispectral index (BIS)-guided (target range 40–60) or to a weight-related (2 mg.kg^− 1^) manual administration of propofol for induction of general anesthesia. The primary endpoint was the incidence of hypotension following the administration of propofol. Secondary endpoints included the degree of hypotension and correlations between BIS and drop in mean arterial pressure (MAP). Incidences were analyzed with Fisher’s Exact-test.

**Results:**

Of the 240 patients enrolled into this study, 235 predominantly non-geriatric (median 48 years, 25th – 75th percentile 35–61 years) patients without severe concomitant disease (88% American Society of Anesthesiology physical status 1–2) undergoing ear, nose and throat surgery, ophthalmic surgery, and dermatologic surgery were analyzed. Patients who were manually administered propofol guided by BIS (*n* = 120) compared to those who were given propofol by weight (*n* = 115) did not differ concerning the incidence of hypotension (44% vs. 45%; *p* = 0.87). Study groups were also similar regarding the maximal drop in MAP compared to baseline (33% vs. 30%) and the proportion of hypotensive events related to all measurements (17% vs. 19%). Final propofol induction doses in BIS group and NON-BIS group were similar (1.93 mg/kg vs. 2 mg/kg). There was no linear correlation between BIS and the drop in MAP at all times (*r* < 0.2 for all) except for a weak one at 6 min (*r* = 0.221).

**Conclusion:**

Results of our study suggest that a BIS-guided compared to a weight-adjusted manual administration of propofol for induction of general anesthesia in non-geriatric patients will not lower the incidence and degree of arterial hypotension.

**Trial registration:**

German Registry of Clinical Trials (DRKS00010544), retrospectively registered on August 4, 2016.

**Electronic supplementary material:**

The online version of this article (10.1186/s12871-018-0522-8) contains supplementary material, which is available to authorized users.

## Background

Propofol is a commonly used intravenous agent for induction and maintenance of general anesthesia.

Animal studies have clearly demonstrated that propofol decreases blood pressure in a dose-dependent manner [1, 2]. The administration of propofol in a dose recommended for induction (1.5–2.5 mg/kg) has been reported since its clinical introduction to be frequently associated with arterial hypotension [3–6].

The prevention of arterial hypotension is an important part of anesthetic management due to possible causal effects of arterial hypotension on morbidity and mortality [7–11].

The bispectral index (BIS) is an electroencephalogram (EEG)-derived parameter to monitor the hypnotic effects of anesthetics. BIS was shown to correlate well with the level of sedation produced by propofol and to accurately predict loss of consciousness [12, 13]. Despite the various limitations inherent to all brain function monitors using processed EEG signals, very recent publications have confirmed the role of the BIS Monitor (Medtronic, Minneapolis, MN, USA) as one of the commercially available standard devices to monitor brain function for depth of anesthesia [14]. Accordingly, use of BIS could allow for individual titration of propofol to a desired hypnotic level and thus might attenuate adverse cardiovascular effects caused by individually overdosing it during induction of general anesthesia.

The main objective of this study was to investigate whether manual administration of propofol guided by BIS compared to a weight-based manual administration for induction of general anesthesia reduces the incidence of arterial hypotension.

## Methods

This was a prospective, randomized, controlled trial conducted in accordance with CONSORT guidelines from February 2012 until April 2013 at Marburg University Hospital. It was approved by the local ethics committee (Medical faculty of Marburg University; approval number: 109/10; 23 August 2010) and registered retrospectively on August 4, 2016 at the German Registry of Clinical Trials (DRKS-ID: DRKS00010544) because registration was not mandatory at the time the study was designed.

Eligible participants were adults scheduled to undergo minor elective surgery (ear, nose and throat surgery, ophthalmic surgery, and dermatologic surgery) under general anesthesia.

Exclusion criteria and information concerning subject randomization and study group allocation in accordance with the most recent CONSORT Statement [15] are described in detail in Additional file 1.

Drugs given orally for premedication shortly prior to patient transfer to the operating rooms were etoricoxib 90 mg and tapentadol prolonged release 100 mg. No benzodiazepines were administered. After arrival in the induction area, pulse oximetry, 3- or 5-lead electrocardiography and non-invasive blood pressure (NIBP) were commenced, baseline vital signs were recorded and an IV cannula was inserted. In addition, a BIS sensor was placed on each patient’s forehead and connected to a BIS Vista monitor using software version 3.0 (Covidien Deutschland GmbH, Neustadt, Germany) with a smoothing rate of 10 s.

**Fig. 1:**
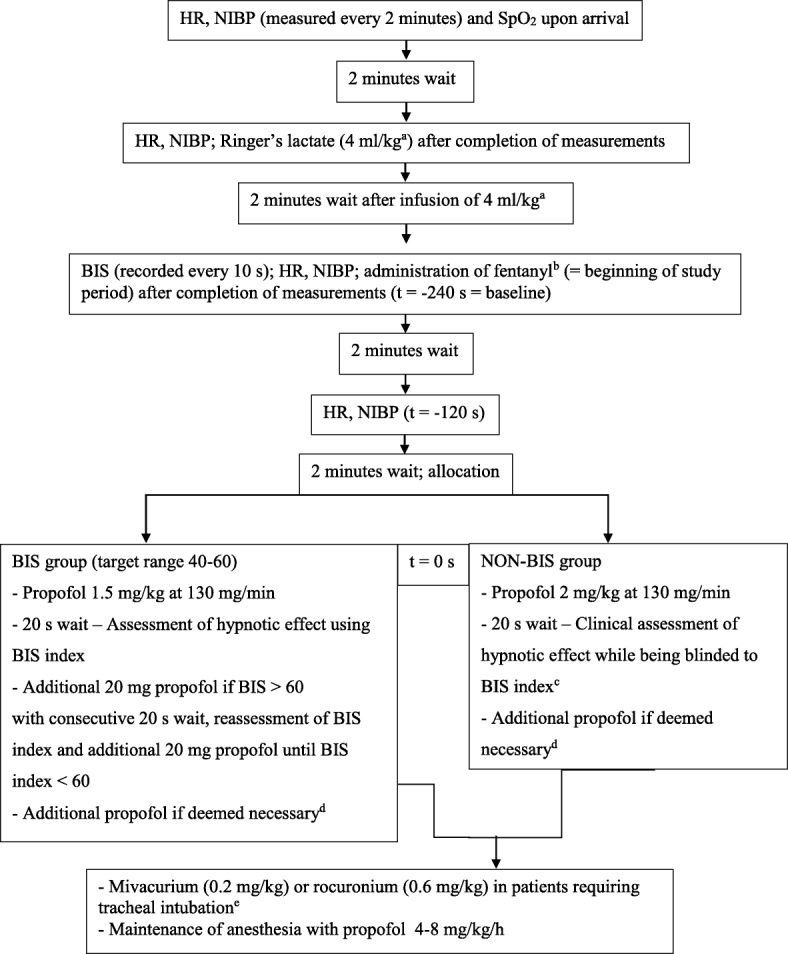
Interventions and measurements included in the study protocol. Legend: HR: Heart rate. NIBP: Non-invasive blood pressure. SpO_2_:Peripheral oxygen saturation measured by pulse oximetry. BIS: Bispectral index. ^a^In patients with a body mass index (BMI) > 25 the volume infused was constrained to the weight corresponding to a BMI of 25, ^b^Fentanyl 0.2 mg and in patients > 70 years or < 55 kg fentanyl 0.1 mg, ^c^At the discretion of the anesthetist usually done by checking the verbal response, the response to light touch and if eyelash reflex is abolished, ^d^Dose of additional propofol at the discretion of the anesthetist, ^e^Neuromuscular blocking agents were applied at the discretion of the anesthetist

Before induction of anesthesia all patients were administered 4 ml/kg Ringer’s lactate. Induction of anesthesia was started with fentanyl followed by propofol (propofol 1% MCT, Fresenius Kabi, Bad Homburg, Germany) applied by an IV infusion pump that was also used for maintenance of anesthesia (Fig. 1). After insertion of the airway, patient’s lungs were ventilated with 30–50% oxygen in air. During the study period, defined as the first 12 min following the time when fentanyl was given, a maximum of 50 ml of crystalloid infusion was given. Drugs administered during the study period were constrained to propofol, fentanyl, mivacurium, rocuronium and a vasopressor (theoadrenaline plus cafedrine - Akrinor®). Both pressure threshold and measures (i.e. head-down tilting and/or vasopressors) of rescue treatment of hypotension as well as the dose of the vasopressor were at the discretion of the assigned anesthetist.

Data recorded by members of the study team (D.N. and S.T.) for each study subject included age, gender, weight, height, ASA physical status, concomitant diseases, regular medication, kind of surgery, type of airway, rescue treatment for hypotension, and amount of propofol, fentanyl, mivacurium, and rocuronium administered. NIBP, heart rate (HR) and BIS-index were recorded as outlined in Fig. 1. In addition, time to loss of eyelash reflex, time to placement of airway and success of placement of airway were also recorded.

### Outcome measures

The aim of this study was to compare the manual administration of propofol by weight (2 mg/kg) to the manual administration of propofol guided by BIS (target range 40–60) concerning its hypotensive effects. To date there is no consensus on the definition of hypotension [16–18]. We defined hypotension as mean arterial pressure (MAP) < 60 mmHg in accordance with one of the many definitions used to study intraoperative hypotension.

The primary endpoint of this study was the number (proportion) of patients with hypotension. Blood pressure was measured every 2 min during the study period.

Secondary endpoints were: the degree of hypotension (difference between baseline MAP and each subsequent measurement of MAP (ΔMAP)) at each subsequent time of measurement, the degree of maximal hypotension (maximal difference between baseline MAP and lowest MAP (Δ MAP_max_)) at any subsequent time of measurement, the rate of hypotensive vs. normotensive MAP measurements, the number (proportion) of patients with severe hypotension (MAP < 50 mmHg), the number (proportion) of patients administered vasopressors and number (proportion) of patients in whom Trendelenburg-positioning was performed at the discretion of the anesthetist to treat a drop in blood pressure, the number (proportion) of patients with hypertension (MAP > 140 mmHg), the number (proportion) of patients with tachycardia (HR > 100/min), and the correlation between BIS-index and MAP.

In addition, we carried out several post hoc analyses. First, we performed a post-hoc sensitivity analysis considering excluded patients “all hypotensive” and “all not hypotensive”. Second, we investigated the impact of various patient characteristics on the maximal relative decrease in MAP in all patients. Details concerning the sensitivity analysis and the analysis concerning the impact of patient characteristics on decrease in MAP are described in Additional file 1. Third, we examined hypotension with an alternative endpoint using a systolic blood pressure < 80 mmHG to define hypotension.

### Sample size calculation

According to our own clinical experience a decrease in MAP < 60 mmHg occurs in about 50% of patients that undergo induction of anesthesia with propofol. A reduction of the incidence of hypotension from 50 to 30% is considered a clinically relevant success. Based on these assumptions 102 study subjects per group are required to detect a difference when using a double sided Fisher’s exact test with a probability level of 5% and a power of 80%. To compensate for possible drop outs 120 patients per group were scheduled to be enrolled.

### Statistical analysis

Statistical analysis was performed using JMP 12, SAS Institute Inc., Cary, NC and SPSS 22, IBM Corp., Armonk, NY. The Kolmogorov-Smirnov test was used to explore normality of distribution of continuous variables. Given some variables were non-normally distributed, we report continuous data as median, 25 and 75% percentiles. Discrete variables are expressed as number, proportion and 95%-confidence intervals. Incidences were analyzed with the chi^2^-test and the Fisher’s exact test where appropriate. Continuous variables were analyzed with the Mann-Whitney-U test. Strength of correlation between two continuous variables was assessed by Pearson’s correlation coefficient *r*.

## Results

**Fig. 2:**
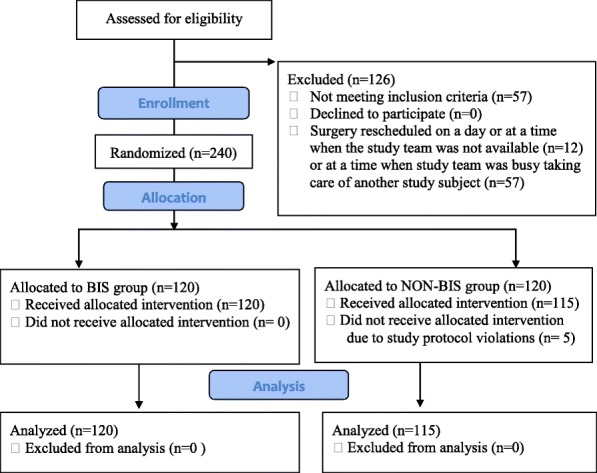
CONSORT study flow diagram

Two hundred forty patients were enrolled over a period of 14 months. Five of those did not receive the allocated intervention because of a violation of the study protocol leaving 235 patients for analysis (Fig. 2).

Patients of BIS group compared to those of NON-BIS group were similar concerning their biometric characteristics, their concomitant medication and various parameters related to anesthesia and surgery (Table 1).

**Table 1:** Patient characteristics and variables related to anesthesia and surgery

	BIS	NON-BIS	*p*-value
	(*n* = 120)	(*n* = 115)	
Gender (male/female)	70/50 (58; 49-67/42; 33-51)	75/40 (65; 56-74/35; 26-44)	0.29
Age (years)	49 (35, 61)	47 (34, 61)	0.85
Height (cm)	174 (168, 180)	176 (168, 182)	0.25
Weight (kg)	78 (70, 89)	82 (71, 90)	0.14
ASA 1	42 (34; 26–43)	49 (43; 33–52)	0.26
ASA 2	65 (55; 45–63)	50 (43; 34–53)	
ASA 3	13 (11; 6–18)	16 (14; 8–22)	
Arterial hypertension	34 (28; 21-37)	38 (33; 25–42)	0.48
Heart failure > NYHAII	0 (0; 0–0)	3 (2.6; 1–7)	0.12
Atrial fibrillation	1 (0.8; 0–5)	2 (1.7; 0–6)	0.62
Ischemic heart disease	4 (3.3; 1–8)	7 (6.1; 2–12)	0.37
Peripheral artery disease	3 (2.5; 1–7)	0 (0; 0–0)	0.25
Diabetes	11 (9.2; 5–16)	10 (8.7; 4–15)	1.00
Dyslipidemia	6 (5; 2–11)	7 (6.1; 2–12)	0.78
β-blockers^a^	16 (13; 6–18)	24 (21; 14–29)	0.16
Antihypertensive drugs^b^	33 (28; 20–36)	39 (34; 25–43)	0.29
ENT surgery	103 (86; 78–92)	94 (82; 73–88)	0.65
Dermatologic surgery	13 (11; 6–18)	15 (13; 7–21)	
Ophthalmic surgery	4 (3; 1–8)	6 (5; 2–11)	
Tracheal tube	104 (87; 79–92)	99 (86; 78–92)	0.90
Laryngeal mask	16 (13; 8–21)	16 (14; 8–22)	
Fentanyl (μg/kg)	2.7 (2.3, 2.9)	2.6 (2.3, 2.9)	0.61
Mivacurium^d^	97 (81; 73–87)	93 (81; 72–88)	0.86
Rocuronium^e^	8 (7; 3–13)	6 (5; 2–11)	
Mivacurium (mg/kg)	0.2 (0.2, 0.3)	0.2 (0.2, 0.2)	0.67
Rocuronium (mg/kg)	0.5 (0.3, 0.5)	0.5 (0.3, 0.5)	0.90
Propofol (mg/kg)^f^	1.93 (1.7, 2.3)	2 (2, 2)	0.74
Propofol airway (mg/kg)^g^	2.06 (1.7, 2.4)	2 (2, 2.5)	0.07
Propofol eyelash reflex (s)^h^	110 (90, 130)	110 (83, 150)	0.6
Airway (s)^i^	370 (320, 420)	380 (310, 430)	0.26
Failed airway^j^	9 (8; 3–14)	6 (5; 2–11)	0.60
Vasopressors^k^	13 (11; 6–18)	14 (12; 7–20)	0.84
Treatment of hypotension^l^	17 (14; 7–19)	15 (13; 7–21)	0.85

Continuous variables are presented as median (25%-, 75%-percentile) and discrete variables are presented as number (proportion; 95%-confidence intervals). BIS Bispectral index. *ASA* Physical status graded according to American Society of Anesthesiologists. *NYHA* Classification of cardiac failure according New York Heart Association. *ENT* Ears, nose and throat. ^a^Patients on β-blockers. ^b^Patients on any antihypertensive medication except for β-blockers. ^c^Patients with any cardiovascular disease in their history. ^d^Patients who were administered mivacurium. ^e^Patients who were given rocuronium. ^f^Induction dose of propofol: until BIS < 60 (Group BIS) or fixed weight-related dose (Group NON-BIS). ^g^Propofol dose administered until placement of the airway. ^h^Time from beginning of propofol injection until abolished eyelash reflex. ^i^Time from beginning of propofol injection until placement of the airway excluding patients with failed airway at first attempt. ^j^Patients in whom placement of the airway was not successful at first attempt. ^k^Patients who were administered vasopressors. ^l^Patients who were administered vasopressors, patients in whom head-down tilting was performed and patients who underwent both antihypotensive treatments

**Fig. 3:**
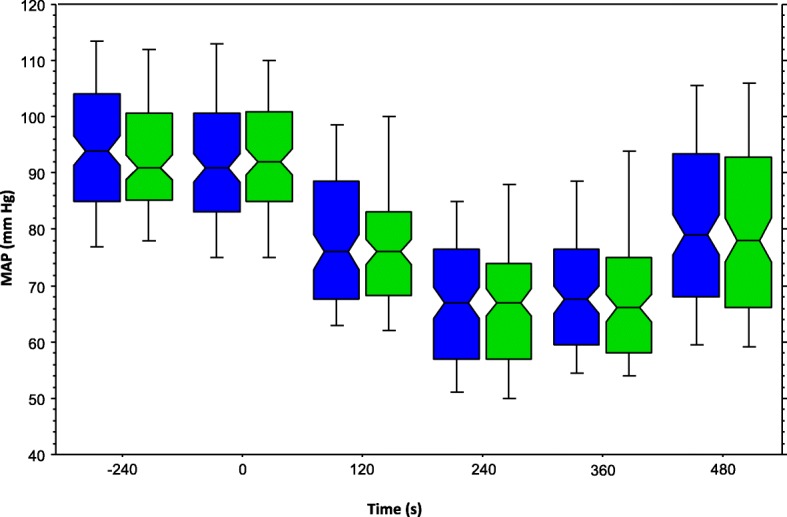
Mean arterial pressure over time. Legend: Box-plot of mean arterial pressures of BIS group (blue) and NON-BIS group (green) during the study period. While the bottom and the top of the box represent the first and third quartile, whiskers represent the 10th and the 90th percentile. The line in the middle of the box indicates the median and the notches of the box show the 95% confidence interval of the median

There was no significant difference (*p* = 0.90) between both study groups concerning the proportion of patients with at least one hypotensive event. Both study groups were also similar with respect to the proportion of patients with at least one severe hypotensive event, the proportion of hypotensive events and severe hypotensive events related to all measurements, the degree of drop in MAP at all measuring times, and the maximal decrease in MAP. Study groups differed concerning the proportion of patients with at least one hypertensive or tachycardic event and the proportion of hypertensive or tachycardic events related to all measurements following injection of propofol (Table 2 and Fig. 3).

**Table 2:** Hemodynamic parameters

	BIS	NON-BIS	*p*-value	summary statistics^a^
	(*n* = 120)	(*n* = 115)		
MAP at 120 s vs. BL (%)	82 (75, 91)	83 (75, 92)	0.44	1
MAP at 240 s vs. BL (%)	71 (62, 80)	73 (65, 80)	0.23	2
MAP at 360 s vs. BL (%)	71 (64, 80)	73 (62, 84)	0.50	2
MAP at 480 s vs. BL (%)	85 (72, 98)	85 (73, 103)	0.60	0
Hypotension^b^	53 (44; 35–54)	52 (45; 36–55)	0.90	0.98 (0.74–1.30)
Severe hypotension^b^	12 (10; 5–17)	16 (14; 8–22)	0.42	0.72 (0.36–1.45)
Hypotension rate^c^	82/479 (17; 14–21)	86/460 (18; 15–23)	0.55	0.91 (0.70–1.20)
Severe hypotension rate^c^	17/479 (3; 2–6)	20/460 (4; 3–7)	0.22	0.82 (0.43–1.54)
Max. MAP drop^d^ (mmHg)	33 (24, 44)	30 (21, 41)	0.26	3
	BIS	NON-BIS	p-value	summary statistics^a^
Max. MAP drop^d^ (%)	35 (26, 43)	33 (25, 43)	0.39	2
Minimal MAP (mmHg)	61 (55, 70)	62 (54, 69)	0.98	1
Hypertension^b^	0 (0; 0–2.5)	4 (3; 1–9)	0.22	0.11 (0.01–1.97)
Tachycardia^b^	4 (3; 1–8)	16 (14; 8–22)	0.01	0.24 (0.08–0.71)
Hypertension rate^c^	0/479 (0; 0–0.6)	4/460 (1; 0–2)	0.06	0.18 (0.06–1.99)
Tachycardia rate^c^	4/479 (1; 0–2)	18/460 (4; 2–6)	< 0.01	0.22 (0.08–0.65)

Data are median (25%-, 75%-percentile) or numbers (proportion; 95% confidence interval). ^a^Data are median differences or RR and 95% CI

*BIS* Bispectral index. *MAP* Mean arterial pressure. *BL* Baseline mean arterial pressure. ^b^Number of patients with that particular event at least once after administration of propofol. ^c^Proportion of this event related to all measurements following the administration of propofol, ^d^Baseline MAP (MAP prior to administration of fentanyl) minus lowest MAP in the study period

**Fig. 4:**
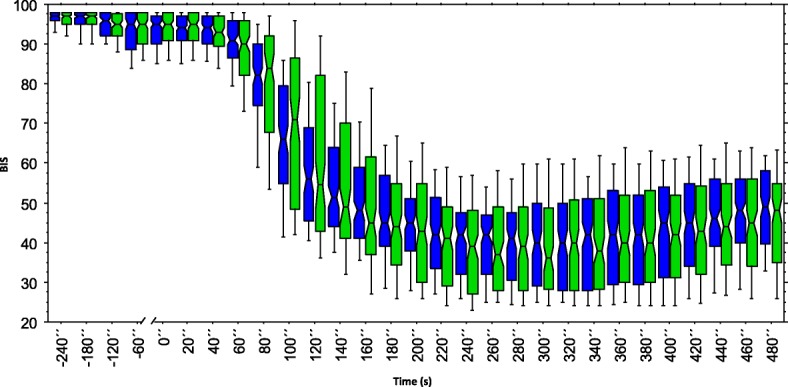
BIS-indices over time. Legend: Box-plot of BIS- indices of BIS group (blue) and NON-BIS group (green) during the study period. While the bottom and the top of the box represent the first and third quartile, whiskers represent the 10th and the 90th percentile. The line in the middle of the box indicates the median and the notches of the box show the 95% confidence interval of the median

BIS-indices recorded every 10 s were similar in patients of the BIS group compared to those in the NON-BIS group during the course of the study period (Fig. 4) consistent with similar doses of propofol administered (Table 1).

Correlations between the drop in BIS on the one hand and the drop in MAP and the degree of decrease in MAP on the other hand following administration of propofol are presented in Additional file 2: Table S3. There were no linear correlations except for a very weak one between BIS and MAP at 6 min after administration of propofol.

Results of a post hoc stepwise backward linear regression analysis to examine the impact of various variables on maximal relative drop in MAP revealed that age was by far the strongest predictor. While some antihypertensive drugs were also found to have a significant impact, all other variables tested were found to have no significant effects (Additional file 3: Table S4).

## Discussion

Hemodynamic stability is an important goal of patient management in anesthesia. Therefore, we studied the hypothesis that a BIS-guided manual administration compared to a weight-related manual administration of propofol would reduce the incidence of arterial hypotension during induction of general anesthesia.

Results of our study could not confirm this hypothesis. In addition, all other secondary outcome parameters we used to examine the hypotensive effects of propofol were similar in both study groups. Results of a post-hoc analysis using systolic blood pressure as an alternative endpoint with a pressure < 80 mmHG as a threshold to define hypotension could not confirm our hypothesis either. Interestingly, there was even a trend for patients in the BIS group to have a higher incidence of hypotension with the drop in blood pressure being more pronounced (Additional file 4: Table S2B).

Hypertension and Tachycardia were observed less frequently in the BIS group. Whether a BIS-guided induction could prevent hypertension and tachycardia needs to be studied in a future trial as these events were only secondary endpoints in our study.

To date, this is the first clinical trial to study if a manually-titrated, BIS-guided vs. a weight-related administration of propofol can reduce arterial hypotension. The rationale for this study is based on the idea that arterial hypotension which is frequently observed during induction of general anesthesia with propofol might be caused to some degree by an individual overdose and that the use of BIS monitoring allows for administration of propofol tailored to its hypnotic effects in individual patients which could prevent the administration of an overdose.

The sensitivity of an individual patient to the hypnotic effects of propofol varies. In the first place it depends on body weight as reflected by the common practice of a weight-related administration. Studies also unequivocally demonstrated that age is another important factor in this respect with elderly patients needing less propofol than young patients [19, 20]. Results of these dose finding studies were confirmed by results of our linear regression analysis demonstrating that age was the strongest predictor of drop in MAP compared to all variables tested. Both pharmacodynamic and pharmacokinetic reasons account for the age-dependent sensitivity to propofol [21, 22]. These include a lower initial distribution volume (volume of the central compartment) resulting in higher propofol concentrations right after injection [23] and a decrease in cardiac output in elderly patients [24]. The latter is underpinned by studies which have demonstrated an inverse relationship between cardiac output and plasma propofol concentrations [25, 26]. Accordingly, cardiac diseases that go along with a reduced cardiac output are of particular importance for patient’s sensitivity to propofol during induction. This observation is confirmed by results of a very recent study comparing the efficacy of anesthetic depth control using closed-loop vs. TCI administration of propofol guided by BIS in predominantly non-geriatric patients with an average ejection fraction < 40% [27]. Unlike age and cardiac function, the role of gender concerning patient’s sensitivity to propofol is less clear [28, 29]. Given the various factors that have an impact on patient’s sensitivity to propofol, it is impossible to predict the exact requirement of a patient for propofol. By analogy, it is conceivable that some patients who are administered propofol by weight are administered an overdose which intensifies the depressant effects of propofol to the cardiovascular system given propofol’s hypotensive effects were demonstrated to be dose dependent [1, 2].

Earlier studies that investigated the dose requirements and the side effects of propofol used clinical endpoints such as loss of consciousness or loss of eye lash reflex [19, 30, 31]. Using a clinical endpoint such as loss of consciousness clearly allows detecting the minimal dose of any drug with hypnotic effects. However, it does not rule out an overdose as any deeper levels of anesthesia cannot be detected with this endpoint. On the contrary, use of an electroencephalogram (EEG)-derived depth of anesthesia monitor enables to detect an overdose and more importantly in this context the extent of an overdose. BIS indices have been shown to correlate with propofol target concentrations and the level of sedation produced by propofol and to accurately predict loss of consciousness [12, 13, 32, 33]. Consistent with these results, BIS has been applied successfully as a target parameter to guide induction and maintenance of general anesthesia both using closed-loop delivery and manually controlled administration of propofol [34].

Accordingly, use of the BIS-monitor facilitates the individual titration of propofol to a desired hypnotic level and thereby might reduce the incidence and extent of unintended side-effects such as arterial hypotension. However, so far, no study has been published confirming this assumption by comparing the effects of a manually controlled BIS-guided to a weight-based administration of propofol on blood pressure during induction. In contrast, a study comparing closed-loop controlled administration of propofol using bispectral index as the controlled variable with manually controlled administration of propofol found that the maximal drop in blood pressure during induction was more pronounced in patients who were manually given propofol [35]. The unavailability of commercially available medical devices that are certified for use in patients constitutes, however, a massive barrier which impedes the closed loop technology to be implemented in daily clinical practice. It was, therefore, our intention to study our hypothesis with certified devices that have a CE mark and that are used in everyday clinical practice.

In our study the patients belonging to the BIS group were administered an initial bolus of 1.5 mg/kg followed by increments of 20 mg until BIS index was < 61. This regimen resulted in similar doses of propofol administered to both study groups consistent with similar BIS indices in both study groups. We chose to administer 1.5 mg/kg as an initial bolus as this is the lower limit of the dose recommended for induction of general anesthesia (1.5–2.5 mg/kg) by the manufacturer. This is in accordance with an early study using a clinical endpoint (unconsciousness detected by loss of verbal contact with the patient) to identify the smallest effective doses of propofol for induction [19]. An initial bolus of 1.5 mg/kg is also consistent with results of a very recent multicenter trial comparing the feasibility and efficacy of an automated (closed-loop) to a manual BIS-guided administration of propofol under similar conditions (non-geriatric patients, 2 μg/kg fentanyl prior to propofol) [36]. Median [IQR] propofol induction doses in the closed-loop and the manual group were 1.4 (1.2–1.8) and 1.8 (1.6–2.2), respectively. In this context it is important to note that these patients had been given lorazepam 1–2 mg orally as premedication while patients of our study had not been given any sedatives prior to induction. In accordance with an initial bolus of 1.5 mg/ kg were findings of another very recent clinical trial that also studied propofol requirements in unpremedicated non-geriatric patients [37]. Mean required propofol doses (95% confidence intervals) during closed-loop anesthesia induction were 2.06 mg/kg (1.68–2.43) in the hypnosis group vs. 1.79 mg/kg (1.54–2.03) in the non-hypnosis group. In contrast, an initial bolus of 1.5 mg/kg is inconsistent with findings of a prospective cohort comparison between non-geriatric obese and lean patients using a BIS-guided closed-loop co-administration of propofol and remifentanil [38]. Median (IQR) propofol induction dose in unpremedicated obese and lean patients were 1.2 (1.1–1.6) and 1.3 (1.0–1.7), respectively. Results of that latter study suggest that 1.5 mg/kg as an initial bolus in the BIS group might have led to administering similar induction doses in both groups of this study.

Interestingly, results of this study demonstrated that no clear-cut linear correlations between MAP and BIS following administration of propofol exist suggesting that arterial hypotension in the observed range is not associated with low BIS-indices. These findings are consistent with results of another study which demonstrated that lower BIS values in patients administered propofol manually guided by BIS compared to BIS-guided closed-loop administration of propofol were not associated with different minimal MAP between study groups [36]. Accordingly, results of our study also suggest that the use of a BIS monitor and probably any other EEG-based monitor to titrate administration of propofol to an adequate level of anesthesia does not significantly reduce the incidence and degree of arterial hypotension in non-geriatric patients.

This conclusion is consistent with results of a recent clinical trial in which the hemodynamic effects of a manual BIS-guided induction of anesthesia with propofol compared to etomidate were studied [39]. Despite the BIS-guided protocol, 8 out of 23 patients (35%) given propofol for induction had arterial hypotension defined as a MAP < 55 mmHg. Interestingly, the incidence of hypotension observed in that study is similar to the one seen in the present study despite a lower mean dose of propofol (1.14 mg/kg vs. 1.93 mg/kg) and a much lower infusion rate (0.5 mg/kg/min vs. 130 mg/min). Lower infusion rates have consistently been shown to be associated with reduced dose requirements and prolonged induction times [19, 31, 40–42]. However, results of previous clinical trials which didn’t use BIS to guide administration of propofol to study the impact of infusion rates of propofol on arterial blood pressure have been inconsistent [19, 30, 31, 40–43]. In this context, a very recent study in non-geriatric patients evaluating the feasibility and efficacy of automated versus manually controlled BIS-guided induction with propofol is remarkable [36]. Results of that study showed that different infusion rates (approximately 30 mg/min vs 60 mg/min) did not result in a different minimal MAP during induction suggesting that propofol infusion rates for induction of anesthesia may not have a substantial impact on arterial blood pressure in non-geriatric patients. Considering the uncertainty about the impact of speed of injection on decrease in arterial blood pressure, in this study propofol was infused at the same rate (130 mg/min) in both groups.

The prevention of arterial hypotension is of particular relevance to anesthetic care given the negative effects arterial hypotension may have on patient outcome. A case-control study conducted among 48,241 patients showed a significant association between intraoperative hypotension and the risk of a postoperative stroke within 10 days after surgery [9]. More recently, results of a retrospective analysis in 33,330 patients undergoing non-cardiac surgery indicated that even a short duration (< 5 min) of a MAP < 55 mmHg is associated with an increased risk for both AKI and myocardial injury [10]. Therefore, even short lasting periods of arterial hypotension that frequently occur following induction of general anesthesia with propofol should be avoided.

This study has several limitations related to 1) the drop-outs, 2) the bispectral index, and 3) the study protocol.

1) There were 5 drop-outs who all belonged to the NON-BIS group. A post hoc sensitivity analysis considering all 5 patients to have the lowest MAP at the different time points showed that the severe hypotension rate was significantly higher in the NON-BIS group and that there was a trend for the hypotension rate to be significantly higher in the NON-BIS group (Additional file 5: Table S2C).

2) All devices using processed EEG including BIS have various limitations inherent to their technology [14] which can result in incorrectly assessing the depth of anesthesia. We chose a target range of 40–60 in accordance with the recommendations of the manufacturer, the results of studies examining propofol requirements for induction and maintenance [35, 44], and the findings of trials investigating the efficacy of BIS to prevent awareness [45]. While the upper threshold is supported by good evidence, this is not the case for the lower limit (BIS < 40) which we used to define an overdose. Accordingly, we cannot rule out that for some patients a BIS < 45 or even < 50 would have been the correct individual threshold to define an overdose. However, results of our study presented in Fig. 4 indicate that a higher threshold wouldn’t have produced a different result concerning the similarities between both study groups with respect to drop in bispectral indices.

3) BIS indices plotted over time showed that the overall distribution of BIS-indices including those < 40, < 45 and < 50 were similar in both groups consistent with similar doses of propofol administered to patients of both groups. This means that the administration of propofol in the BIS-group consisting of an initial 1.5 mg/kg bolus followed by 20 mg boluses with a 20 s wait in between each administration of propofol until BIS was < 61 did not result in patients in the BIS-group being administered lower doses of propofol and thereby having a lower incidence of overdose when using a BIS < 40 as a threshold for propofol overdose.

We chose to stop the injection of propofol once BIS was < 61 as did Möller Petrun and Kamenik in their study [39]. There is a time delay between injection of the hypnotic and decrease in BIS index which results from drug transition time (from injection site to effect site) and from index calculation time. Using simulated signals, the latter was shown for decreasing BIS indices (from 98 down to 52) to be 14 s [46]. Adding another few seconds for drug transition, 20 s wait in between propofol injections were considered by us to be sufficient to avoid overdosing propofol. However, considering the huge variability in time delay observed with smaller decreasing BIS-indices (decrease of 11 or 12 index points) ranging from 15 to 66 s [46], 20 s wait may have been too short to avoid overdosing propofol in some patients. A longer wait in between the injections was felt unsuitable though for standard anesthetic practice.

For the same reason, a relatively high infusion rate was chosen, as this was shown in many studies to result in faster induction times [19, 30, 31, 40, 41]. High infusion rates compared to low infusion rates result in higher propofol plasma concentrations. Accordingly, the dose of propofol in transit (from the injection site to the effect site) that will cause the BIS to further decrease after stopping administration of propofol is the higher, the faster the speed of injection of propofol. Even though results of studies examining the impact of propofol infusion rates on drop in arterial blood pressure including more recent results of studies that used closed-loop administration with BIS as the target have been inconsistent, we cannot rule out a negative impact of the high infusion rate in our study on arterial hypotension as it was higher than the ones of most other studies [36, 39].

Therefore, a higher target BIS (e.g. 65 instead of 60) combined with a longer wait in between the repetitive propofol injections (e.g. every 30 s instead of every 20 s), a lower infusion rate (e.g. 100 mg/min instead of 130 mg/min), and a smaller initial bolus of propofol (e.g. 1 mg/kg instead of 1.5 mg/kg) and might have resulted in patients of the BIS group to get lower doses of propofol and thereby to have significantly less BIS values < 40 compared to patients in the NON-BIS group.

However, it is important to remember in this context that there was no linear correlation between BIS indices and MAP suggesting that even if neither patient in the BIS group had had a BIS < 40, no significant differences concerning the drop in MAP between the study groups would have been observed.

Participating patients in our study were predominantly non-geriatic and without significant cardiac comorbidity. Given the impact of age and cardiac output on patient’s requirement for propofol it is conceivable that we could have observed lower BIS values and a more pronounced drop in MAP in patients of the NON-BIS group had we carried out the study in geriatric patients with low cardiac output.

## Conclusions

Results of this study suggest that use of BIS monitoring to guide manual propofol administration following intravenous fentanyl for induction of general anesthesia in non-geriatric patients does not reduce the dose of propofol administered and thereby the incidence and the degree of arterial hypotension compared to a weight-related (2 mg/kg) administration of propofol when using an initial bolus of 1.5 mg/ kg combined with a comparably high infusion rate (130 mg/min). Future trials studying the impact of a BIS-guided induction with propofol on arterial blood pressure in geriatric patients and patients with low cardiac output seem reasonable.

## Additional files

Additional file 1: **Methods**. Details concerning 1) Exclusion criteria, patient consent, randomization, and allocation, 2) Post hoc analyses: A- Sensitivity analysis considering excluded patients “all hypotensive” and “all not hypotensive”; B- Stepwise linear regression analysis to study the impact of various patient characteristics on the maximal relative decrease in MAP in all patients. (DOCX 16 kb)

Additional file 2: **Table S3.** Correlations between BIS and MAP and ΔMAP. (DOCX 13 kb)

Additional file 3: **Table S4.** Stepwise linear regression analysis of impact of patient characteristics on decrease in MAP. (DOCX 14 kb)

Additional file 4: **Table S2B.** Hemodynamic data with systolic blood pressure as endpoint (DOCX 17 kb)

Additional file 5: **Table S2C.** Hemodynamic parameters assuming all 5 excluded patients were hypotensive. (DOCX 16 kb)

## Abbreviations

ASA: American Society of Anesthesiologists
BIS: Bispectral index
BL: Baseline
CI: Confidence interval
EEG: Electroencephalogram
ENT: Ears, nose and throat
HR: Heart rate
IQR: Interquartile range
MAP: Mean arterial pressure
NIBP: Non-invasive blood pressure
NYHA: New York Heart Association
RR: Relative risk
SD: Standard deviation
SpO_2_: Peripheral oxygen saturation measured by pulse oximetry
TCI: Target controlled infusion
