# Supplementary material for: Bispectral index to guide induction of anesthesia: a randomized controlled study
Source: BMC Anesthesiol. 2018 Jun 15;18:66. doi: 10.1186/s12871-018-0522-8 (PMC6003112; doi:10.1186/s12871-018-0522-8)
Supplement: Supplementary file 1 — Methods. Details concerning 1) Exclusion criteria, patient consent, randomization, and allocation, 2) Post hoc analyses: A- Sensitivity analysis considering excluded patients “all hypotensive” and “all not hypotensive”; B- Stepwise linear regression analysis to study the impact of various patient characteristics on the maximal relative decrease in MAP in all patients. (DOCX 16 kb) [file 12871_2018_522_MOESM1_ESM.docx]

**Exclusion criteria, patient consent, randomization, and allocation**

Exclusion criteria were participation in another study, pregnancy and lactation, any medical condition that would require rapid sequence induction, American Society of Anesthesiologists (ASA) physical status > 3, any disease with a potential for compromised alertness (due to underlying disease or sedative premedication), and unavailability of the study team on the day the procedure was scheduled to take place.

Patients eligible for inclusion were identified during the pre-anesthesia visit by a member of the study team and enrolled by physicians of the study team (D.R., C.A., L.E., H.W.) after having obtained written informed consent. A randomization list was generated in advance using the internet site <http://www.randomnumbers.info/> by a member of the departmental study section who was not involved in this study. A non-balanced randomization sequence was chosen in order to ensure allocation concealment given the open label study design. Opaque envelopes were sealed and numbered after having inserted a sheet of paper with the corresponding group allocation (BIS or Standard of care = NON-BIS). Right before induction of anesthesia the envelope with the smallest number was opened by a member of the study team (D.N., S.T.) in order to randomly allocate the study subject in accordance with the most recent CONSORT Statement [15] to one of the study groups.

**Details concerning post hoc analyses**

1. Sensitivity analysis considering excluded patients “all hypotensive” and “all not hypotensive”. For the “all hypotensive analysis”, each of the 5 excluded patients was assigned the lowest MAP minus 1 mmHg observed at the different time points in the NON-BIS group. To calculate the relative drop in MAP compared to baseline, all 5 patients were assigned the median baseline MAP of their group. In contrast, for the “all not hypotensive analysis”, each of the 5 excluded patients were assigned the highest MAP observed at the different time points in their group. Second, we investigated the impact of various patient characteristics (Table 4) on the maximal relative decrease in MAP in all patients. These variables were subjected to a stepwise linear regression analysis using a backward and a forward ex-/inclusion technique. In each step the least significant factor was eliminated if p was greater than 0.05. The quality of the final regression model was judged using the amount of explained variance of the model and by graphically checking if the standardized residuals were normally distributed and applying the Shapiro–Wilk test as a statistical confirmation. The Durbin–Watson statistic (a value between 0 and 4 with an optimum of 2.0), leverage plots as a graphical tool, and the variance inflation factor (VIF; optimal value: 1.0) were used as indicators of autocorrelation or collinearity of parameters included in the model. Interactions between these factors were investigated using graphical tools (interaction profiles plots) offered by the statistical package used for data analysis (JMP 8.0.1; SAS Institute Inc., Cary, NC, USA).

We found no evidence for relevant autocorrelation. Durbin-Watson statics was 2.02 and the autocorrelation coefficient -0.017 detects as non-significant (p>0.58). VIF was 1.26. The same final model was produced using either the forward or the backward procedure indicating the stability of the model (r2 = 0.21; r2 corrected: 0.196; p<0.0001).
