# Supplementary material for: Bispectral index to guide induction of anesthesia: a randomized controlled study
Source: BMC Anesthesiol. 2018 Jun 15;18:66. doi: 10.1186/s12871-018-0522-8 (PMC6003112; doi:10.1186/s12871-018-0522-8)
Supplement: Supplementary file 2 — Table S3. Correlations between BIS and MAP and ΔMAP. (DOCX 13 kb) [file 12871_2018_522_MOESM2_ESM.docx]

**Table 3** Correlations between BIS and MAP and ΔMAP

120 s 240 s 360 s 480 s

BIS-MAP 0.167 0.190 0.245 0.173

BIS-ΔMAP 0.044 0.110 0.221 0.170

Data are Pearson´s correlation coefficients (*r*) between BIS and MAP and ΔMAP at the four times when blood pressure was measured following the injection of propofol.

BIS: Bispectral index. MAP: Mean arterial pressure. ΔMAP: Difference between baseline MAP and MAP at the particular time. BIS-MAP: Correlations between BIS and MAP at the particular times. BIS-ΔMAP: Correlations between BIS and ΔMAP at the particular times.
