# Supplementary material for: Bispectral index to guide induction of anesthesia: a randomized controlled study
Source: BMC Anesthesiol. 2018 Jun 15;18:66. doi: 10.1186/s12871-018-0522-8 (PMC6003112; doi:10.1186/s12871-018-0522-8)
Supplement: Supplementary file 3 — Table S4. Stepwise linear regression analysis of impact of patient characteristics on decrease in MAP. (DOCX 14 kb) [file 12871_2018_522_MOESM3_ESM.docx]

**Table 4** Stepwise linear regression analysis of impact of patient characteristics on decrease in MAP

Step Variable Removed from r-square after removal model at p = of variable

1 Cardiac disease 0.999 0.298

2 ASA 2 vs 3 0.918 0.298

3 Diuretics 0.922 0.298

4 Gender 0.896 0.227

5 BMI 0.603 0.226

6 Height 0.588 0.224

7 BIS vs. NON-BIS 0.538 0.222

8 Weight 0.449 0.220

9. Airway 0.347 0.217

10. CCB 0.315 0.209

11. ASA 1 vs. 2&3 0.140 0.196

Final model β-blockers 0.022

AT1 antagonist 0.016

ACE inhibitors 0.011

Age <0.0001

ACE inhibitor: Angiotensin-converting-enzyme inhibitor. ASA: American Society of Anesthesiologists. ASA 1/2/3: physical status according to classification of American Society of Anesthesiologists. AT1 antagonist: Angiotensin II receptor (subtype 1) antagonist. BIS: Bispectral index. BMI: Body mass index. CCB: Calcium channel blocker. MAP: Mean arterial pressure. Table 4 shows the results of the stepwise elimination of non-significant variables. The final model consists of four variables presented at the bottom of the table. According to the final model, the prediction formula for the percentage decrease in MAP is as follows: 10.09% + age x 0.33 + ACE-inhibitors x 7.69 + AT1 antagonists x 7.96 + β-blockers x 5.89.
