# Supplementary material for: Bispectral index to guide induction of anesthesia: a randomized controlled study
Source: BMC Anesthesiol. 2018 Jun 15;18:66. doi: 10.1186/s12871-018-0522-8 (PMC6003112; doi:10.1186/s12871-018-0522-8)
Supplement: Supplementary file 4 — Table S2B. Hemodynamic data with systolic blood pressure as endpoint (DOCX 17 kb) [file 12871_2018_522_MOESM4_ESM.docx]

**Table 2B** Systolic blood pressure as endpoint

BIS NON-BIS p-value (n=120) (n=115)

SAP Baseline (mmHg) 133 (119, 149) 129 (119, 144) 0.31

SAP at 120 s (mmHg) 108 (97, 125) 110 (99, 122) 0.94

SAP at 240 s (mmHg) 97 (81, 107) 84 (84, 110) 0.56

SAP at 360 s (mmHg) 94 (81, 107) 96 (83, 113) 0.21

SAP at 480 s (mmHg) 107 (96, 128) 111 (92, 125) 0.77

SAP at 120 s vs. BL (%) 84 (75, 93) 86 (77, 94) 0.42

SAP at 240 s vs. BL (%) 70 (61, 81) 76 (63, 83) 0.08

SAP at 360 s vs. BL (%) 69 (60, 81) 75 (62, 86) 0.07

SAP at 480 s vs. BL (%) 84 (68, 95) 86 (72, 100) 0.41

Hypotension (<80mmHg)^a^ 101 (84; 76-90) 90 (78; 70-85) 0.12

Hypotension rate^b^ 265/480 (55; 51-60) 229/460 (50; 45-54) 0.10

Max. SAP drop^c^ (mmHg) 34 (25, 45) 31 (21, 43) 0.11

Max. SAP drop^c^ (%) 34 (25, 45) 39 (21, 43) 0.12

Minimal SAP (mmHg) 86 (78, 98) 86 (76, 102) 0.25

Hypertension (>180mmHg)^a^ 0 0

Continuous variables are presented as median (25%-, 75%-percentile) and discrete variables are presented as numbers (proportion; 95% confidence interval). BIS: Bispectral index. SAP: Systolic arterial pressure. BL: Baseline systolic arterial pressure prior to administration of fentanyl. ^a^ Number of patients with that particular event at least once after administration of propofol, ^b^ Proportion of this event related to all measurements following the administration of propofol, ^c^ Baseline SAP minus lowest SAP in the study period.
