# Supplementary material for: Bispectral index to guide induction of anesthesia: a randomized controlled study
Source: BMC Anesthesiol. 2018 Jun 15;18:66. doi: 10.1186/s12871-018-0522-8 (PMC6003112; doi:10.1186/s12871-018-0522-8)
Supplement: Supplementary file 5 — Table S2C. Hemodynamic parameters assuming all 5 excluded patients were hypotensive. (DOCX 16 kb) [file 12871_2018_522_MOESM5_ESM.docx]

**Table 2C** Hemodynamic parameters assuming all 5 excluded patients were hypotensive

BIS NON-BIS p-value Summary statistics*
 (n = 120) (n = 120)

MAP at 120 s vs. BL (%) 82 (75, 91) 83 (74, 92) 0.85 1.2

MAP at 240 s vs. BL (%) 71 (62, 80) 73 (63, 80) 0.55 2.0

MAP at 360 s vs. BL (%) 71 (64, 80) 73 (61, 83) 0.47 1.9

MAP at 480 s vs. BL (%) 85 (72, 98) 84 (68, 101) 0.44 0.4

Hypotension^a^ 53 (44; 35-54) 57 (48; 38-57) 0.61 0.930 (0.706-1.225)

Severe hypotension^a^ 12 (10; 5-17) 21 (18; 11-26) 0.10 0.571 (0.295-1.109)

Hypotension rate^b^ 82/479 (17; 14-21) 106/480 (22; 18-26) 0.06 0.777 (0.600-1.007)

Severe hypotension rate^b^ 17/479 (3; 2-6) 40/480 (8; 6-11) < 0.0001 0.425 (0.244-0.739)

Max. MAP drop^c^ (mmHg) 33 (24, 44) 31 (21, 42) 0.50 2

Max. MAP drop^c^ (%) 35 (26, 43) 33 (25, 44) 0.79 2

Minimal MAP (mmHg) 61 (55, 70) 61 (53, 69) 0,5591 0

Data are median (25%- and 75%-percentile) or numbers (proportion; 95% confidence interval). * Data are median differences or relative risk and 95% confidence interval. BIS: Bispectral index. MAP: Mean arterial pressure. BL: Baseline mean arterial pressure. ^a^ Number of patients with that particular event at least once after administration of propofol, ^b^ Proportion of this event related to all measurements following the administration of propofol, ^c^ Baseline MAP (MAP prior to administration of fentanyl) minus lowest MAP in the study period.
